# Bevacizumab reduces cerebral radiation necrosis due to stereotactic radiotherapy in non-small cell lung cancer patients with brain metastases: an inverse probability of treatment weighting analysis

**DOI:** 10.3389/fimmu.2024.1399613

**Published:** 2024-08-27

**Authors:** Jingwei Zhang, Jiayi Yu, Dan Yang, Leilei Jiang, Xin Dong, Zhiyan Liu, Rong Yu, Huiming Yu, Anhui Shi

**Affiliations:** ^1^ School of Basic Medical Sciences, Capital Medical University, Beijing, China; ^2^ Key Laboratory of Carcinogenesis and Translational Research (Ministry of Education/Beijing), Department of Radiation Oncology, Peking University Cancer Hospital and Institute, Beijing, China

**Keywords:** bevacizumab, radiation, radionecrosis, stereotactic, NSCLC, brain metastases

## Abstract

**Background:**

Cerebral radiation necrosis (RN), a severe complication of stereotactic radiotherapy (SRT), has been shown to significantly decrease patient survival time and quality of life. The purpose of this study was to analyze whether bevacizumab can prevent or reduce the occurrence of SRT-induced cerebral RN in non-small cell lung cancer (NSCLC) patients with brain metastases.

**Materials and methods:**

We retrospectively reviewed the clinical records of NSCLC patients with brain metastases from March 2013 to June 2023 who were treated with SRT. Patients were divided into two groups: those in the bevacizumab group received SRT with four cycles of bevacizumab, and patients in the control group received SRT only. Inverse probability of treatment weighting (IPTW) was performed based on a multinomial propensity score model to balance the baseline characteristics. The chi-square test was used. A Cox model was used to evaluate overall survival (OS).

**Results:**

A total of 80 patients were enrolled, namely, 28 patients in the bevacizumab group and 52 patients in the control group. The possibility of developing cerebral RN and/or symptomatic edema (RN/SE) was significantly decreased in patients treated with bevacizumab compared to those who did not receive bevacizumab before IPTW (p=0.036) and after IPTW (p=0.015) according to chi-square analysis. The IPTW-adjusted median OS was 47.7 months (95% CI 27.4-80.8) for patients in the bevacizumab group and 44.1 months (95% CI 36.7-68.0) (p=0.364) for patients in the control group.

**Conclusion:**

The application of bevacizumab concurrent with SRT may prevent or reduce the occurrence of cerebral RN in NSCLC patients with brain metastases.

## 1 Introduction

Lung cancer is most often detected in stage IV when it has metastasized via blood and lymphatic vessels (1). Approximately 40% of non-small cell lung cancer (NSCLC) patients develop brain metastases, with appreciable morbidity and mortality (2). The efficacy of chemotherapy for brain metastasis is mostly poor (3); thus, treating brain metastases in patients with NSCLC is challenging, and a multidisciplinary approach is needed to control intracranial disease effectively.

With advancements in the treatment paradigm of stereotactic radiotherapy (SRT), this technique has become the usual clinical treatment of choice for cerebral metastases (4). Although SRT is an effective and noninvasive strategy for treating cerebral metastases (5), cerebral radiation necrosis (RN) is a common complication in patients after SRT with an occurrence of 5% to 25% and should be given more attention (6–9).

The mechanisms of cerebral RN are still under investigation. One commonly accepted mechanism involves the vascular system. Vascular endothelial growth factor (VEGF), usually considered an angiogenic factor plays an important role in abnormal neovascularization, which promotes tumor progression. An abnormal and disordered vessel structure with high capillary permeability leads to the development of brain edema, which ultimately deteriorates into cerebral RN (10, 11).

According to existing mechanisms, many clinical treatments have been used including bevacizumab treatment. Recent studies have focused mainly on the efficacy and safety of bevacizumab treatment for brain necrosis and have confirmed that bevacizumab remains the most thoroughly characterized and most widely used angiogenesis inhibitor across a range of advanced cancers with poor prognosis (12). In addition, the incidence of cerebral RN is irreversible; thus, treatment with bevacizumab can relieve patients’ cerebral RN symptoms and improve their quality of life but not cure cerebral RN (11). There is little research on the use of bevacizumab as a precautionary measure to reduce the toxicity of irradiation in patients with NSCLC to minimize the occurrence of RN. Thus, we designed this study to compare the outcomes of NSCLC patients with brain metastases treated with SRT concurrent with 1 cycle of bevacizumab and followed by 3 continuous cycles of bevacizumab with the outcomes of those treated with SRT only. To our knowledge, this is the first study to explore whether bevacizumab can prevent the occurrence of SRT-induced cerebral RN. This study shed a light on the prevention of cerebral RN which may provide patients with an improvement of quality of life with fewer adverse reaction.

## 2 Materials and methods

### 2.1 Eligibility criteria

We retrospectively reviewed the clinical records of NSCLC patients with brain metastases from March 2013 to June 2023 treated with SRT. This retrospective study was approved by the Institutional Review Board (IRB) of Peking University Cancer Hospital & Institute. Informed consent was exempted by the IRB due to the retrospective nature of this research. All methods were performed in accordance with relevant guidelines and regulations. Patient records were anonymized and deidentified before analysis of the data.

The inclusion criteria for the study were defined as follows: (1) histologically or cytologically confirmed NSCLC; (2) age 18 years or older; (3) Karnofsky Performance Status (KPS) score of ≥70; (4) 10 or fewer cerebral metastases confirmed by computed tomography (CT) or magnetic resonance imaging (MRI); and (5) SRT administered for cerebral metastases.

Eighty patients were divided into two groups according to the application of bevacizumab: 28 patients in the bevacizumab group received SRT and four cycles of bevacizumab, and 52 patients in the control group received SRT only.

### 2.2 SRT technique

**Figure 1:**
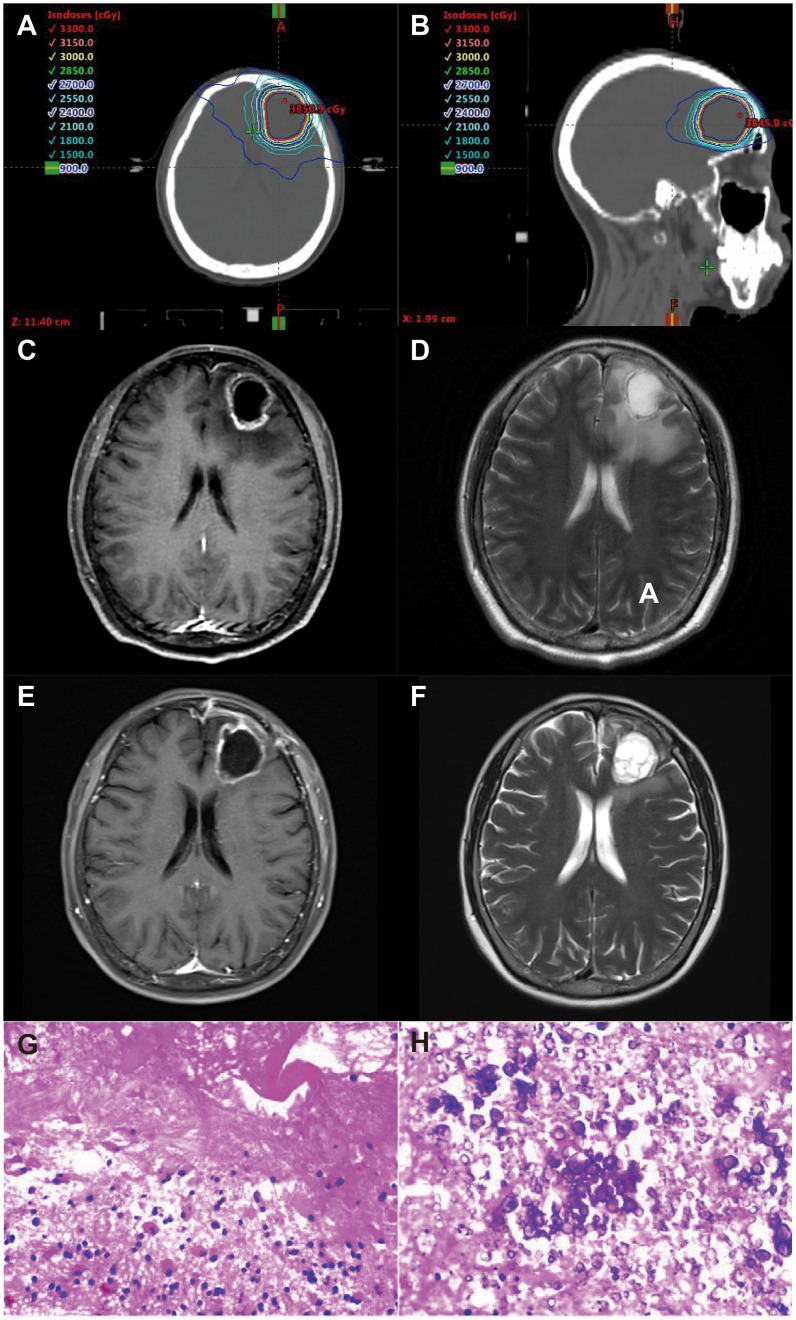
61 year old, female patient with non-small cell lung cancer treated with SRT without bevacizumab. Representative axial **(A)** and sagittal **(B)** plane of a VMAT plan of SRT treatment [PTV, 30 Gy isodose line (yellow)]. Representative axial planes in CE-T1 and T2 MRI sequences before SRT **(C, D)** and two months after surgical resection **(E, F)** with symptomatic radiation necrosis resulting in dizziness and headache. **(G, H)** representative pathological staining images diagnosis of radiation necrosis.

In all patients, SRT was delivered using the volumetric-modulated arc radiotherapy (VMAT) technique. All treatment plans were designed with the Eclipse treatment planning system based on a 6-MV photon beam from a Varian linear accelerator (True Beam or Edge). All patients underwent a simulated computed tomography scan, fusion into thin layers, contrast-enhanced T1-weighted MRI. The gross tumor volume (GTV) was determined based on T1-weighted axial-enhanced MRI, occasionally guided by positron emission tomography, and was expanded with an additional 2-mm margin to determine the planning target volume (PTV). Most patients were administered a short-term prophylactic course of dexamethasone after SRT. Based on the specific size and location of the tumor, the PTV was prescribed at a dose of 18-24 Gy in 1 fraction, 27-30 Gy in 3 fractions or 30 Gy in 5 fractions. Organs at risk comprised the brain tissue and brainstem. The maximum dose to the brainstem was less than or equal to 10 Gy, conditionally suggesting that limiting the volume to 12 Gy was less than or equal to10 cm^3^ of a single segmentation of brain tissue. A representative example is given in **Figure 1**.

The organs at risk comprised the brain stem, eyes, hippocampus, lens and optic nerve. The brain stem delineates the entire brain stem within the scanning range and increases it by 3mm to form the PRV. The maximum brain stem dose did not exceed 31Gy. As for eyes, the mean eyes dose did not exceed 20Gy and the maximum eyes dose did not exceed 35Gy. The maximum dose to optic nerve and optic chiasma was less than 22.5Gy for both sides. The volume of the lens is exposed to a dose less than 6Gy.

### 2.3 Bevacizumab treatment

A total of 4 cycles of bevacizumab were administered: the first cycle involved a dosage of 7.5 mg/kg bevacizumab administered once every 3 weeks, with the first dose on the same day as the first SRT fraction; following SRT, 3 additional cycles of bevacizumab were administered. The time of administration was more than 90 min for the first treatment and more than 60 min for each subsequent treatment. Patients were monitored by electrocardiogram throughout the administration process to closely observe their reactions to the medication.

### 2.4 Diagnosis of RN

Pathological examination is a highly reliable diagnostic method for distinguishing local tumor recurrence from cerebral RN. However, it is difficult to carry out in clinical practice (13, 14) for several reasons. First, there are many important functional areas or cranial base structures around intracranial tumors that are hard to resect surgically or biopsy stereotactically. In addition, few patients consent to biopsy after SRT. Moreover, the results of pathological examination can only reflect local lesions rather than showing overall pathological changes. Finally, conducting surgical resection or stereotactic biopsy contradict the goals of SRT, which are to increase survival time and improve patient quality of life.

The presumption of a symptomatic RN was based on the occurrence of adverse events of at least CTCAE v5.0 grade 2 (Common Terminology Criteria for Adverse Events). The suspicion of RN was based on typical MRI morphological findings on contrast-enhanced (CE) T1 sequences, which included the appearance of a spreading wavefront, spread to the contralateral hemisphere and/or multiple foci with discrete contrast enhancement only, and/or dynamic 18F-FET PET findings showing increasing time activity curves specifically in cases of RN (15). A stereotactic, usually PET-guided, biopsy was performed in specific cases to eliminate the possibility of tumor progression and to ascertain the suspicion of RN. The identification of necrotic tissue, absence of viable tumor tissue, and low proliferation indices (Ki-67) labeling index within the range of 1%) provided strong evidence supporting the diagnosis of RN. The structural and molecular imaging results and histological findings were examined by relevant experts and presented and dissected during multidisciplinary treatment (MDT) team meetings. A representative example is given in **Figure 1**. Patients who did not exhibit typical signs of either RN or tumor progression but continued to experience high levels of symptoms, such as severe headache, drowsiness, and paresis, for more than 6 weeks after SRT due to persistent symptomatic edema (SE) despite steroid treatment (as evidenced by T2-weighted follow-up MRI results) were categorized as SE patients. All patients with an SE diagnosis had to exhibit clinical stabilization/improvement over time to exclude tumor progression. The diagnosis of SE was also resolved by an MDT team. Patients with imaging-diagnosed edema or pathologically diagnosed cerebral RN (RN/SE) were included in the study.

In addition, dose diffusion magnetic resonance imaging (DWI) as an auxiliary method was used to differentiate RN from tumor recurrence, which present similar characteristics in standard MR images (16). DWI is able to acquire a signal for the movement of water protons in cellular spaces of the body, which can describe normal and histopathological characteristics. The apparent diffusion coefficient (ADC) value, including qualitative methods that are either restricted or facilitated and quantitative methods, is a sensitive method for detecting cerebral RN (17). Low levels on DWI and high ADC values suggest metastasis and RN, whereas high levels on DWI and low ADC values suggest tumor recurrence.

### 2.5 Outcomes

Patients’ cerebral edema index (EI), brain necrosis incidence rate, and difference in overall survival (OS) time were observed, and the toxicity and side effects of bevacizumab were evaluated with further exploration of their mechanism. Local control (LC) rate was defined as the time after SRT treatment until local failure.

### 2.6 Statistical methods

IBM SPSS 20.0 software and R were used to analyze the data. Measurement data are described as the mean ± standard deviation or the median (quartile) and were analyzed with a rank-based nonparametric method. Enumeration data are described as the frequency, relative frequency, and constituent ratio and were tested with the chi-square test and Fisher’s precision probability test. We used propensity score matching to select and pair the exposed group with controls according to their basic conditions and symptoms before treatment. Multifactorial logistic regression analysis and generalized linear models were used for clinical data analysis to control for potential confounding factors. The curves for OS were plotted using the Kaplan−Meier method. The log-rank method and Cox proportional hazard model were used to conduct single-factor and multivariate analyses. Bonferroni correction was used for multiple comparisons.

To minimize the effects of potential confounding factors between the two treatment groups to ensure the reliability and rigor for different clinical outcomes, inverse probability of treatment weighting (IPTW) based on a multinomial propensity score model was used. We used a logistic regression model to estimate the multinomial propensity including the following potential confounders: sex, age, smoking status, tumor lymph node metastasis (TNM) stage and mutation type. To assess the covariate balance in the IPTW sample, standardized mean differences in covariate values were applied. The covariables were sex classified as male or female; age classified as ≥60 or age <60 years old; smoking status classified as yes or no; TNM stage classified as stage III or stage IV; and EGFR mutation classified as yes or no. Both IPTW-adjusted and unadjusted Kaplan−Meier estimates of OS rates are presented. For sensitivity analyses, doubly robust IPTW analysis using the Cox model was performed to adjust for potential residual confounding. Significance was set to p=0.05.

## 3 Results

### 3.1 Patient demographics

We identified 109 lesions in 80 NSCLC patients with a pathological diagnosis of brain metastasis whose characteristics are shown in **Table 1**. There were 46 men and 34 women with a median age of 66 years (range, 27-85 years). Epidermal growth factor receptor (EGFR) mutation was observed in 37 (46.25%) patients, which was close to half of the patients; 1 (1.25%) patient had a KRAS mutation, 1 (1.25%) patient had a BRAF mutation, 7 (8.75%) patients had an ALK mutation, 4 (5.00%) patients had nononcogenic driver mutations, and 29 (36.25%) patients had no genetic mutation.

**Table 1:** Patient demographics and baseline clinical characteristics of patients.

	Unweighted, number(%)				Weighted, mean(%)			
Characteristic	Overall (n=80)	Bevacizumab-used(n=28)	Bevacizumab-unused(n=52)	P	Bevacizumab-used	Bevacizumab-unused	P	test SMD
Age				0.453			0.906	0.030
Median(range)	66(27-85)	65.5(40-85)	66(27-85)					
≥60	23(28.8%)	10(35.7%)	13(25.0%)		72.2	70.9		
<60	57(71.2%)	18(64.3%)	39(75.0%)		27.8	29.1		
Sex				0.029			0.924	0.025
Female	34(42.5%)	17(60.7%)	17(32.7%)		43.5	42.3		
male	46(57.5%)	11(39.3%)	35(67.3%)		56.5	57.7		
Smoking				1.000			0.735	0.091
Yes	30(37.5%)	10(35.7%)	20(38.5%)		41.8	37.3		
No	50(62.5%)	18(64.3%)	32(61.5%)		58.2	62.7		
TNM stage				1.000			0.728	0.096
I	9(11.3%)	3(10.7%)	6(11.5%)					
II	11(13.8%)	3(10.7%)	8(15.4%)					
III	21(23.3%)	3(10.7%)	18(34.6%)		19.7	16.0		
IV	39(48.8%)	19(67.9%)	20(38.5%)		80.3	84.0		
Gene mutation				0.009			0.979	0.007
EGFR	37(46.3%)	19(67.9%)	18(34.6%)		45.3	45.0		
ALK	7(8.8%)	1(3.6%)	6(11.5%)					
BRAF	1(1.3%)	0(0.0%)	1(1.9%)					
KRAS	1(1.3%)	0(0.0%)	1(1.9%)					
Non-non-oncogenic driver	4(5.0%)	3(10.7%)	1(1.9%)					
NA	29(36.3%)	5(17.9%)	24(46.2%)		54.7	55.0		

Twenty-nine patients had received first-line treatment, and 10 had received second-line chemotherapy. The most commonly used chemotherapeutic agents were carboplatin (n=31), pemetrexed (n=23), etoposide (n=11), and albumin-bound paclitaxel (n=10). Twenty-eight patients were treated with 1 cycle of bevacizumab concurrent with SRT and 3 continuous cycles of bevacizumab after SRT, and 52 patients who were not administered bevacizumab after SRT were included as the control group. The median tumor size is 2.5cm (0.8-14.6 cm). Over all, the median gross tumor volumes (GTV) is 5.6 cm^3^ (0.2-38.5 cm^3^) and the median planning target volumes (PTV) is 14.2 cm^3^ (1.0-85.1 cm^3^). The median prescribed does and fraction (BED) is 56Gy (42.9-84.6Gy).

### 3.2 Correlation of RN/SE with the application of bevacizumab before and after IPTW

Bevacizumab was relatively well tolerated by those who received it during and after SRT, and these patients have, to date, exhibited a lower incidence of cerebral RN up to now. Only 1 patient administered bevacizumab as a precautionary measure had biopsy-proven cerebral RN, while 8 patients had symptomatic edema (SE), and 3 patients had pathologically diagnosed cerebral RN. The incidence of RN was significantly decreased in patients treated with bevacizumab compared to those who did not receive this treatment (p=0.036).

The propensity score model consisted of sex, age, smoking status, tumor lymph node metastasis (TNM) stage and EGFR mutation type. After applying IPTW adjustment, the baseline characteristics were found to be balanced between the bevacizumab and control groups, as shown in **Table 1**. After IPTW, it was found that patients treated with bevacizumab after SRT were significantly less likely (p=0.015) to develop RN/SE.

### 3.3 Survival analyses

**Figure 2:**
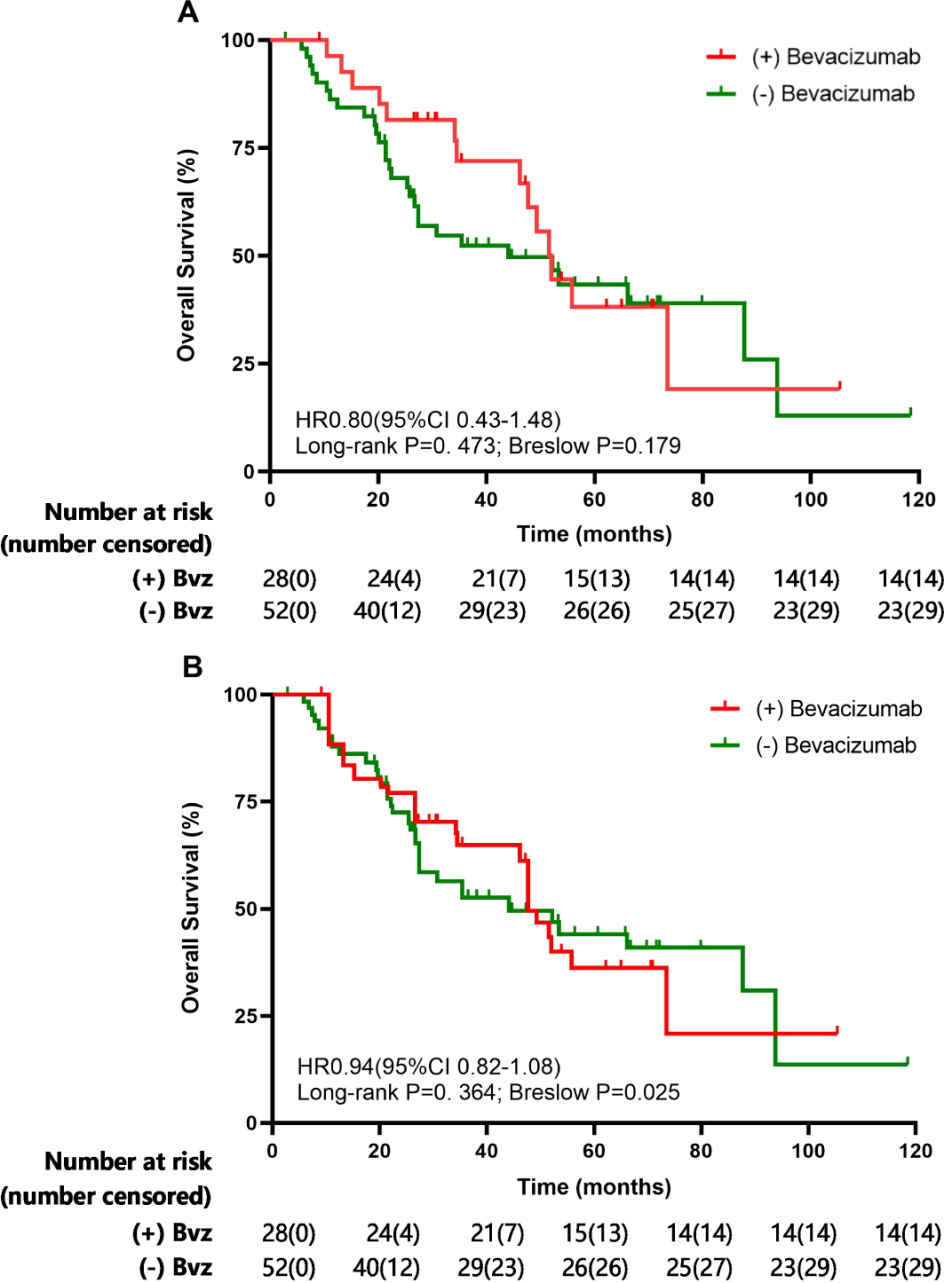
Overall survival (OS) evaluated the Kaplan-Meier method before **(A)** and after **(B)** inverse-probability of treatment weighting analysis for overall cohorts.

The survival data for NSCLC patients who did or did not receive bevacizumab during and after SRT were analyzed. In our study, OS was defined as the time from the date of diagnosis until the date of death from any cause. The median follow-up of the entire cohort was 60.6 months [95% CI 45.1-76.1]. The median OS in patients with NSCLC receiving bevacizumab during and after SRT was 52.0 (95% CI 46.7-57.3) months compared with 44.1 (95% CI 16.5-71.7) months in patients not receiving bevacizumab (p=0.473; **Figure 2A**). The 1- and 3-year OS rates were 92.6% and 61.2%, respectively, in the group of NSCLC patients receiving bevacizumab and 84.3% and 49.8%, respectively, in the group not receiving bevacizumab (**Figure 2A**).

Patients with gene mutations had a significantly higher median OS of 87.7 (95% CI 48.9-125.5) months compared with a median OS of 21.4 (95% CI 15.3-27.5) months for patients without gene mutations (p<0.001). Among gene mutation patients, the median OS of 87.7 months (95% CI 49.9-125.5) in the bevacizumab group was higher than the median OS of 52.0 months (95% CI 44.2-59.8) in the control group, but the difference was not significant (p=0.333).

### 3.4 IPTW analysis of survival outcomes

The IPTW-adjusted median OS was 47.7 months (95% CI 27.4-80.8) for patients who received bevacizumab during and after SRT and 44.1 months (95% CI 36.7-68.0) (p=0.364) for patients who received SRT only. As shown in **Figure 2**, the comparison of bevacizumab group or control group shown no clear OS benefit.

### 3.5 Prognostic model

The influencing factors for RN/SE analyzed with the Cox regression model are presented in **Table 2**.

**Table 2:** Multivariate analyses of prognostic factors related to OS before propensity score matching.

Variables	RE/SE-free survival univariate	Hazard ratio	95%-CI
BEV treatment	P=0.078	0.159	0.020-1.232
Sex	P=0.035	0.457	0.221-0.945
Age	P=0.544	0.816	0.422-1.575
Smoking	P=0.794	1.101	0.534-2.273
TNM	P=0.011	1.629	1.117-2.376
Mutation	P<0.001	0.183	0.086-0.387

Bevacizumab treatment was not considerably associated with prolonged OS (HR, 0.159; 95% CI: 0.020-1.232; p=0.078) but was still a protective factor. Gene mutation (HR, 0.183; 95% CI: 0.086-0.387; p<0.001) and sex (HR, 0.457; 95% CI: 0.221-0.945; p=0.035) were protective factors that were significantly related to prolonged OS. Tumor lymph node metastasis (TNM) stage (HR, 1.629; 95% CI: 1.117-2.376; p=0.011) was a risk factor for prolonged OS.

### 3.6 Response

The median follow-up time was 43.2 months (range, 3.1-127.3 months) for the entire cohort. Overall, 28 patients and 43 lesions in the bevacizumab group and 52 patients and 66 lesions in control group were evaluated for best response. The partial response (PR), stable disease (SD) and progressive disease (PD) rate were 32.5%, 55.0% and 12.5%, respectively. At the end of follow-up, 1 patient in bevacizumab group and 11 patients in control group have experienced RN. The disease control rate is 87.5%. The local control rate for the patients in bevacizumab group and control group at 1 year was 88.9% and 84.3%, respectively.

### 3.7 Treatment-related toxicities

The use Bevacizumab was well tolerated in this study. The acute toxicities (defined as toxicity occurring between the start of treatment and up to 3 months after the completion of treatment and assessed according to the National Cancer Institute Common Terminology Criteria for Adverse Events version 5.0) were summarized in **Table 3** and there was no late toxicity (more than 3 months after the completion of treatment and assessed according to the observed at the National Cancer Institute Common Terminology Criteria for Adverse Events version 5.0) end of follow-up. 40 patients (50.0%) experienced grade 1-2 toxicities and only 4 patients (2.5%) experienced grade 3 toxicities. Overall, Leukopenia (32.5%) was most commonly seen in this cohort. Hypertension (22.5%), Headache (20.0%) and Neutropenia (20.0%) were commonly seen.

**Table 3:** Treatment-related acute toxicities.

	Bevacizumab group (n=28)				Control group (n=52)			
	Grade 1 No. (%)	Grade 2 No. (%)	Grade 3 No. (%)	Grade 4-5 No. (%)	Grade 1 No. (%)	Grade 2 No. (%)	Grade 3 No. (%)	Grade 4-5 No. (%)
Leukopenia	12(15.0)	7(8.8)	2(2.5)	0(0.0)	4(5.0)	1(1.3)	0(0.0)	0(0.0)
Neutropenia	8(10.0)	2(2.5)	1(1.3)	0(0.0)	2(2.5)	2(2.5)	1(1.3)	0(0.0)
Thrombocytopenia	2(2.5)	1(1.3)	0(0.0)	0(0.0)	3(3,8)	1(1.3)	0(0.0)	0(0.0)
Anemia	6(7.5)	3(3.8)	0(0.0)	0(0.0)	2(2.5)	0(0.0)	0(0.0)	0(0.0)
Elevated AST	0(0.0)	0(0.0)	0(0.0)	0(0.0)	0(0.0)	0(0.0)	0(0.0)	0(0.0)
Elevated ALT	4(5.0)	0(0.0)	0(0.0)	0(0.0)	0(0.0)	0(0.0)	0(0.0)	0(0.0)
Headache	8(10.0)	2(2.5)	0(0.0)	0(0.0)	4(5.0)	2(2.5)	0(0.0)	0(0.0)
Fatigue	3(3.8)	0(0.0)	0(0.0)	0(0.0)	3(3.8)	0(0.0)	0(0.0)	0(0.0)
Nausea	2(2.5)	0(0.0)	0(0.0)	0(0.0)	3(3.8)	0(0.0)	0(0.0)	0(0.0)
Hypertension	10(12.5)	4(5.0)	0(0.0)	0(0.0)	3(3.8)	1(1.3)	0(0.0)	0(0.0)
Proteinuria	4(5.0)	1(1.3)	0(0.0)	0(0.0)	0(0.0)	0(0.0)	0(0.0)	0(0.0)
Gastrointestinal perforation	0(0.0)	0(0.0)	0(0.0)	0(0.0)	0(0.0)	0(0.0)	0(0.0)	0(0.0)
Thromboembolism	0(0.0)	0(0.0)	0(0.0)	0(0.0)	0(0.0)	0(0.0)	0(0.0)	0(0.0)

ALT, alanine aminotransferase; AST, aspartate aminotransferase.

## 4 Discussion

The commonly use of SRT in the treatment of brain metastases in NSCLC patients proved to be a usual and effective option (18). However, 5% to 25% patients after SRT will experience cerebral RN which is one of the main limiting toxicities and severely influences patient survival time and quality of life. Bevacizumab now is the most thoroughly characterized and most widely used angiogenesis inhibitor across a range of advanced cancers with poor prognosis. There are many studies focusing on the efficacy and safety of bevacizumab to manage SRT-induced cerebral RN while there is little research focusing on preventing the occurrence of cerebral RN. The purpose of this study was to explore whether bevacizumab can prevent the occurrence of SRT-induced cerebral RN in patients with NSCLC.

We note several important findings in this study. First, bevacizumab can decrease the toxicity of irradiation and prevent the development of cerebral RN in NSCLC patients with brain metastases. Second, a clear OS benefit could not be demonstrated between bevacizumab group and control group. Third, the use of bevacizumab as a precautionary measure is safe in clinical application.

Cerebral RN, a severe complication of SRT, has severe symptoms like headache, epilepsy, cognitive disorder and so on which severely decrease patient quality of life. Previous studies have reported the frequency of RN after radiotherapy. For instance, an RN incidence rate of 10% to 15% in patients who were diagnosed with malignant gliomas and had survived for more than one year after irradiation has been reported (19). A W Lee et al. reported that the incidence of temporal lobe necrosis in patients with nasopharyngeal carcinoma after 9 months to 16 years of radiotherapy ranged from 1.6% to 22.0% (20). Given the widespread use and assessment of SRT, it is now clear that 2% to 5% of SRT patients will develop symptomatic focal cerebral necrosis, which is lower than the incidence rates noted above but remains a limitation of this treatment (21, 22). In our research, the total probability of biopsy-proven cerebral RN was 5% (4 of 80), while the incidence of SE was 10% (8 of 80).

At present, most clinical studies are based on bevacizumab treatment of refractory brain edema and cerebral RN after SRT (23–26). To our knowledge, this is the first study confirming that bevacizumab concurrent with SRT may prevent or reduce the occurrence of cerebral RN in NSCLC patients with brain metastases. Patients’ quality of life and overall survival are both important. Though our result didn’t show clear benefit of prolonging OS, improving NSCLC patients’ quality of life was also essential.

The safety of bevacizumab is equally important to its efficacy. In our study, patients experienced few adverse reactions. Puyuan Xing et al. (27) confirmed that a first-line regimen containing bevacizumab in terms of safety assessment showed acceptable adverse reactions and better effectiveness than a non-bevacizumab regimen in patients with NSCLC. Ning Tang et al. (28) also reported that compared to chemotherapy alone, the combination of chemotherapy and bevacizumab as first-line and maintenance treatment resulted in improved curative rates and tolerable adverse events in patients with advanced NSCLC. In our study, decreased RN occurrence and manageable adverse events improved patients’ quality of life confirming the efficacy and safety of bevacizumab.

Overall, among all the investigations using bevacizumab as an effective treatment measure for cerebral RN, this study provides a new angle from treatment to prevention. Nevertheless, this study certainly had limitations. First, it was geographically limited to one hospital. Patients from other health care environments may produce different outcomes or confirm our study. Second, the median OS of bevacizumab group and control group was 52.0 months and 44.1 months respectively which is much longer than commonly considered mean median OS of NSCLC patients with brain metastases. The median OS of patients in our study was defined as the time from the date of diagnosis until the date of death from any cause. Thus, the median OS in our study is much longer. Finally, the limited number and the unbalanced proportion of patients assessed may influence our results. To minimize the effects of potential confounding factors between the two treatment groups, inverse probability of treatment weighting (IPTW) based on a multinomial propensity score model was used to perfectly balance essential factors including, sex, age, smoking status, tumor lymph node metastasis (TNM) stage and mutation type.

Despite the limited number of patients assessed and the preliminary nature of the observations presented here, this study initially confirmed the efficacy of bevacizumab to prevent the occurrence of cerebral RN in patients with NSCLC, which may inform the clinical use of SRT for intracranial tumors. In the future, prospective studies and more clinical data are needed to confirm the effectiveness of bevacizumab as a precautionary measure and establish a complete system for the application of bevacizumab to prevent the occurrence of cerebral RN.

## Data Availability

The original contributions presented in the study are included in the article/supplementary material. Further inquiries can be directed to the corresponding author.
